# Comprehensive analysis of cuproptosis-related genes involved in immune infiltration and their use in the diagnosis of hepatic ischemia-reperfusion injury: an experimental study

**DOI:** 10.1097/JS9.0000000000001893

**Published:** 2024-06-27

**Authors:** Xiaopeng Cai, Jingwen Deng, Xiaohu Zhou, Kaiyue Wang, Huiqiang Cai, Yingcai Yan, Jun Jiang, Jia Yang, Jin Gu, Yuan Zhang, Yuan Ding, Qiang Sun, Weilin Wang

**Affiliations:** a Department of Hepatobiliary and Pancreatic Surgery, The Second Affiliated Hospital, Zhejiang University School of Medicine; b Key Laboratory of Precision Diagnosis and Treatment for Hepatobiliary and Pancreatic Tumor of Zhejiang Province; c Research Center of Diagnosis and Treatment Technology for Hepatocellular Carcinoma of Zhejiang Province; d National Innovation Center for Fundamental Research on Cancer Medicine; e Cancer Center, Zhejiang University; f ZJU-Pujian Research & Development Center of Medical Artificial Intelligence for Hepatobiliary and Pancreatic Disease; g Department of Medical Oncology, Sir Run Run Shaw Hospital, School of Medicine, Zhejiang University, Hangzhou; h Department of surgery and International Institutes of Medicine, The Fourth Affiliated Hospital, Zhejiang University School of Medicine, Yiwu, Zhejiang, China; i Department of Medical Oncology, West German Cancer Center, University Hospital Essen, Essen, Germany

**Keywords:** bioinformatics, cuproptosis, hepatic ischemia-reperfusion injury, immune infiltration, machine learning

## Abstract

**Background:**

Hepatic ischemia-reperfusion injury (HIRI) is a common injury not only during liver transplantation but also during major hepatic surgery. HIRI causes severe complications and affects the prognosis and survival of patients. Cuproptosis, a newly identified form of cell death, plays an important role in a variety of illnesses. However, its role in HIRI remains unknown.

**Materials and methods:**

The GSE151648 dataset was mined from the Gene Expression Omnibus (GEO) database, and differences were analyzed for intersections. Based on the differentially expressed genes (DEGs), functional annotation, differentially expressed cuproptosis-related genes (DE-CRGs) identification and lasso logistic regression were conducted. Correlation analysis of DE-CRGs and immune infiltration was further conducted, and DE-CRGs were applied to construct an HIRI diagnostic model. The hierarchical clustering method was used to classify the specimens of HIRI, and functional annotation was conducted to verify the accuracy of these DE-CRGs in predicting HIRI progression. The GSE14951 microarray dataset and GSE171539 single-cell sequencing dataset were chosen as validation datasets. At the same time, the significance of DE-CRGs was verified using a mouse model of HIRI with cuproptosis inhibitors and inducers. Finally, a network of transcription-factor-DE-CRGs and miRNA-DE-CRGs was constructed to reveal the regulation mechanisms. And potential drugs for DE-CRGs were predicted using Drug-Gene Interaction Database (DGIdb).

**Results:**

Overall, 2390 DEGs and 19 DE-CRGs were identified. Through machine learning algorithms, 8 featured DE-CRGs (GNL3, ALAS1, TSC22D2, KLF5, GTF2B, DNTTIP2, SLFN11 and HNRNPU) were screened, and 2 cuproptosis-related subclusters were defined. Based on the 8 DE-CRGs obtained from the HIRI model [area under the curve (AUC)=0.97], the nomogram model demonstrated accuracy in predicting HIRI. Eight DE-CRGs were highly expressed in HIRI samples and were negatively related to immune cell infiltration. A higher level of immune infiltration and expression of CRG group B was found in the HIRI population. Differences in cell death and immune regulation were found between the 2 groups. The diagnostic value of the 8 DE-CRGs was confirmed in the validation of two datasets. The identification of 7 DE-CRGs (SLFN11 excluded) by HIRI animal model experiments was also confirmed. Using hTFtarget, miRWalk and DGIDB database, we predicted that 17 transcription factors, 192 miRNAs and 10 drugs might interact with the DE-CRGs.

**Conclusion:**

This study shows that cuproptosis may occur in HIRI and is correlated with immune infiltration. Additionally, a cuproptosis-related predictive model was constructed for studying the causes of HIRI and developing targeted treatment options for HIRI.

## Introduction

Highlights

- In this paper, we screened 8 cuproptosis-related genes (GNL3, ALAS1, TSC22D2, KLF5, GTF2B, DNTTIP2, SLFN11 and HNRNPU) were associated with HIRI.
- The optimal machine learning model based on the 8 DE-CRGs can accurately predict the risk of HIRI occurrence and assess HIRI subtypes.
- Our findings provided a theoretical basis for the underlying pathophysiological process of cuproptosis and potential HIRI treatments in the future.

Hepatic ischemia-reperfusion injury (HIRI) refers to an important clinical problem seen in patients undergoing trauma, systemic shock and liver surgery (e.g. liver transplantation (LT) and hepatectomy)^1^. HIRI consists of two stages, ischemic insult and reperfusion injury, which lead to hepatic primary necrosis and apoptosis. Furthermore, damaged hepatocytes cause inflammatory cell infiltration and the release of damage-associated molecular patterns, resulting in more hepatocyte damage^2^. With the development of medical treatment and surgical skills, HIRI has mostly occurred in the setting of LT in recent years. In fact, allograft ischemia/reperfusion injury contributes to not only early allograft dysfunction but also chronic graft rejection, and early liver dysfunction can affect 42.9% of HIRI patients after LT^3,4^. Moreover, studies have found that allograft ischemia/reperfusion injury also renders the recipient liver more susceptible to recurrence of tumors, fibrosis and viral hepatitis^5,6^. Despite the considerable efforts made in the therapeutic strategies for HIRI, prevention serves as the main strategy to reduce HIRI^7^. Therefore, there is an urgent need to explore the mechanisms of HIRI and to develop new therapeutics to enhance the efficacy of LT.

Currently, the signal process involved in HIRI-associated hepatocyte death is thought to be apoptosis; however, various cell death signaling pathways have been found to be involved in HIRI pathogenesis, including necroptosis, ferroptosis, autophagy and cell scorching^6,8^. The regulation of cell death is a key strategy for HIRI treatment. Recently, a novel kind of programmed cell death, cuproptosis, was reported in March 2022^9^. Cuproptosis results from mitochondrial dysfunction, which is closely related to the tricarboxylic acid cycle. Cu^2+^ binds to the lipoylated components, with subsequent aggregation of lipoylated mitochondrial enzymes and downregulation of iron-sulfur protein clusters, finally leading to proteotoxic stress and cell death^9^. Copper is a cofactor in various enzymes and is involved in a variety of biological activities, such as iron metabolism, energy metabolism and coagulation^10,11^. Abnormalities in copper lead to different toxic effects. Copper deficiency leads to dysfunction of copper-binding enzymes, which affects normal physiological activities. However, excess copper induces oxidative stress, resulting in cell damage or death^12^. Studies have found that copper is associated with liver diseases. Previous studies have noted that copper deficiency leads to the progression of nonalcoholic fatty liver disease, which results from a decrease in the capacity of the antioxidant system^13^. However, excess consumption of copper leads to experimental hepatotoxicity, which proceeds via the Fenton reaction^14,15^. Another genetic disorder with excess copper is Wilson disease, which is due to excess copper content in the liver^16^. Therefore, we hypothesize that there are some connections between cuproptosis and HIRI, but the precise mechanisms underlying this association are not well understood.

To determine the potential pathogenesis, we mined data from the Gene Expression Omnibus (GEO) database to explore the gene differentiations between normal and HIRI samples. Then, we identified differentially expressed cuproptosis-related genes (DE-CRGs) for multiple bioinformatics analyses. Moreover, we examined the correlation between cuproptosis and immune infiltration in HIRI, which provided a new method to elucidate the mechanism of HIRI. In summary, our study aimed to investigate the contribution of CRGs to the diagnosis and treatment of HIRI.

## Materials and methods

### Data acquisition

Gene expression data of HIRI and non-HIRI samples was mined from the Gene Expression Omnibus database (GEO) (https://www.ncbi.nlm.nih.gov/geo/): the RNA-seq data of GSE151648 (23 preischemic samples and 23 postischemic samples), microarray data of GSE14951 (5 preischemic samples and 5 postischemic samples) and single-cell sequencing data of GSE171539 (1 preprocurement sample and 1 post-reperfusion sample). The original files were background adjusted and quantile normalized by the R package. DEseq2 package offered Variance Stabilizing Transformations to normalize data. If the expression value of multiple probe information corresponded to a gene with the same name, the average value was selected as the expression level of that gene. The CRGs were derived from previous literature, and 347 CRGs were identified^17^.

### Identification of DEGs associated with HIRI

We extracted the preischemic and postischemic DEGs from GSE151648 using the R package “limma”^18^. |log2(FC) | greater than 0.6 and *P* less than 0.05 were chosen as the thresholds to identify DEGs, and the differential analyses were visualized using the R package. Moreover, Gene Ontology (GO) enrichment analysis and Kyoto Encyclopedia of Genes and Genomes (KEGG) pathway analysis were performed using R language.

### Identification and validation of diagnostic genes for DE-CRGs

An intersection of the two gene sets was made using the R package “VennDiagram” between DEGs and CRGs. Then, featured DE-CRGs were screened from the DE-CRGs by least absolute shrinkage and selection operation (LASSO) analysis using the R package “glmnet”^19^. The following parameters were set in the LASSO algorithm: family = “binomial”; alpha = 1; type.measure = “deviance”; and nfolds = 10. Based on the featured DE-CRGs, receiver operating characteristic (ROC) curves of the diagnostic genes were generated using the R package. Furthermore, an extreme gradient boosting model was established based on the featured DE-CRGs, and the ROC curve of this model was generated. In addition, the expression levels of diagnostic genes were verified in GSE14951 and GSE171539.

### Gene set enrichment analysis (GSEA) for featured DE-CRGs

We performed GSEA using the “ClusterProfiler” R package. HIRI samples were classified into high- and low-expression groups based on the cutoff value of the expression levels of featured DE-CRGs. The enriched pathways were sequenced using normalized enrichment scores (NESs). The criterion for significant gene enrichment was *P* less than 0.05.

### Investigation of immune cell infiltration and cell death

Based on the CIBERSORT website (https://cibersortx.stanford.edu/), we used the CIBERSORT algorithm to observe the expression of immune cells in the preischemic and postischemic samples. In addition, Spearman’s correlation analyses between the expression levels of featured DE-CRGs and 22 immune cells were conducted. Next, we determined the association between cell death and featured DE-CRGs using Spearman’s correlation analyses.

### Subcluster analysis with featured DE-CRGs

Using the “ConsensusClusterPlus” R package and the expression profiles of 8 featured DE-CRGs as input information, a hierarchical clustering analysis was performed on the 23 HIRI samples^20^. A principal component analysis (PCA) plot shows the difference between subgroups A and B. In addition, DEG analysis of 2 subgroups of HIRI was performed to describe the biological functions of the featured DE-CRGs. *P* less than 0.05 was used to screen DEGs. “ClusterProfiler” packages in R were used for GO enrichment analysis of DEGs. Finally, we evaluated the immune infiltration and cell death of the 2 subgroups. *P* less than 0.05 was considered to be statistically significant.

### Animals and treatment

The 8-week-old male C57BL/6J mice weighing 20–25 g were acquired from Jiangsu GemPharmatech Company. After a week of adaptation, mice were randomly allocated to 4 groups (*N*=5 each group): the sham, IRI, IRI + ammonium tetrathiomolybdate (ATTM, Sigma-Aldrich, 15060-55-6), IRI + ES–Cu (elesclomol, MCE, 488832-69-5 and CuCl_2_, Sinopharm, 10007818). The IRI models: mice were peritoneally anaesthetized with pentobarbital sodium at 50 mg/kg and then killed by spinal dislocation. The 70% liver ischemia model (left and median liver lobes), in which the portal vein and hepatic artery of the middle and left lobes were blocked, was performed according to previous literature^21^. The hepatic pedicle was blocked for 60 min, and reperfusion times varied after clamp removal. After 6 h of reperfusion, the serum and liver tissues were obtained for further analysis. The mice in the sham model underwent portal vein and hepatic artery isolation with no occlusion. The administration of ATTM: ATTM was administered intravenously (10 mg/kg) 30 min before IRI. At 6 h post-reperfusion, mice were assessed using functional and molecular outcome measurements as former study described^22^. The administration of ES–Cu: mice was injected intraperitoneally with 3 mg/kg of elesclomol and 0.5 mg/kg of CuCl_2_ once every two days, and then was executed after IRI on day 13^23^.

All experimental protocols met the requirements of ARRIVE guidelines, Supplemental Digital Content 2, http://links.lww.com/JS9/C895 (https://arriveguidelines.org) for the reporting of animal experiments^24^.

### Liver injury analysis and quantitative real-time polymerase chain reaction (PCR)

Serum alanine aminotransferase (ALT) and aspartate transaminase (AST) levels were determined and hematoxylin and eosin (H&E) staining of liver tissue was performed to evaluate liver damage. At the same time, we assessed the copper ion content and apoptosis of the liver according to our previous methods^25^. Finally, the total RNA of liver samples was extracted, and the relative mRNA expression of featured DE-CRGs and cytokines was further evaluated. The primers for these genes are listed in Table S1.

### Construction of a transcription factor (TF)-DE-CRGs network and a miRNA-DE-CRGs network

The hTFtarget (http://bioinfo.life.hust.edu.cn/hTFtarget#) is a reliable tool for transcription factors prediction, which has curated comprehensive TF-target regulations from large-scale of ChIP-Seq data of human TFs in 569 conditions^26^. We used the hTFtarget database to predict TFs in liver tissue, which were predicted in at least 2 databases. The miRWalk database ((http://mirwalk.umm.uni-heidelberg.de/) predictes data obtained with a machine learning algorithm including experimentally verified miRNA-target interactions. We used the miRWalk database to predict miRNAs that were targeted at and bound to the -E-CRGs baded on miRanda, TargetScan and miRDB. Finally, we got the regulatory network using Cytoscape software.

### Drug-gene interaction analysis of DE-CRGs

The Drug-Gene Interactions Database (DGIdb, www.dgidb.org) is a web resource that provides information on drug-gene interactions and druggable genes from publications, databases, and other web-based sources^27^. We used the DGIdb database to predict drugs that could interact with the DE-CRGs.

### Statistical analysis

All data are expressed as the mean ± standard deviation. Data processing and statistical analysis were performed using R software. The Wilcoxon rank-sum test or Student’s *t*-test was used to analyze the differences between two groups. The level of statistical significance was set at *P* less than 0.05.

## Results

### Identification of DE-CRGs and functional enrichment analysis

**Figure 1:**
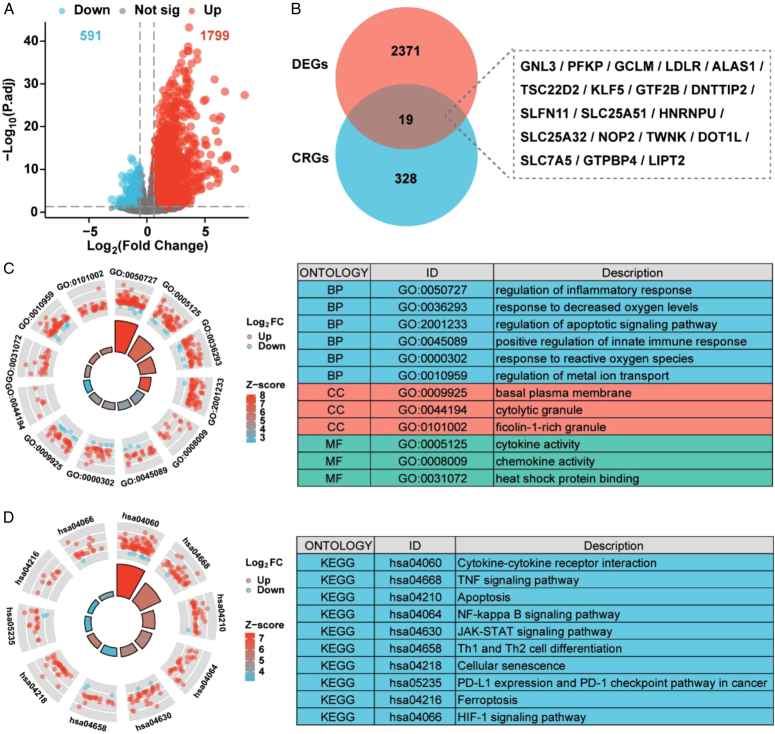
Identification of DE-CRGs and functional enrichment analysis. (A) Volcano plot of HIRI-related DEGs. (B) Intersection between DEGs and CRGs in HIRI. (C) GO enrichment analysis of DEGs in terms of biological processes, cellular components, and molecular functions. (D) KEGG pathway analysis of DEGs. CRGs, cuproptosis-related genes; DEGs, differentially expressed genes; DE-CRGs, differentially expressed cuproptosis-related genes; HIRI, hepatic ischemia-reperfusion injury.

In the GSE151648 dataset, a total of 2390 DEGs (|log2 (FC) | >0.6 and *P* < 0.05) were obtained, of which 591 genes were downregulated and 1799 genes were upregulated in the HIRI group (Fig. 1A). By integrating 2390 DEGs with 347 CRGs, we finally obtained 19 DE-CRGs (Fig. 1B). To investigate the role of these DEGs in HIRI, we conducted a GO/KEGG enrichment pathway analysis. As shown in Fig. 1C, DEGs were mainly enriched in the regulation of inflammatory response, response to reactive oxygen species, regulation of metal ion transport, cytokine/chemokine activity and heat shock protein binding. In addition, the KEGG results showed that cytokine–cytokine receptor interaction, apoptosis, Th1 and Th2 cell differentiation, ferroptosis and the HIF-1 signaling pathway were enriched (Fig. 1D).

### Identification of featured DE-CRGs via machine learning

**Figure 2:**
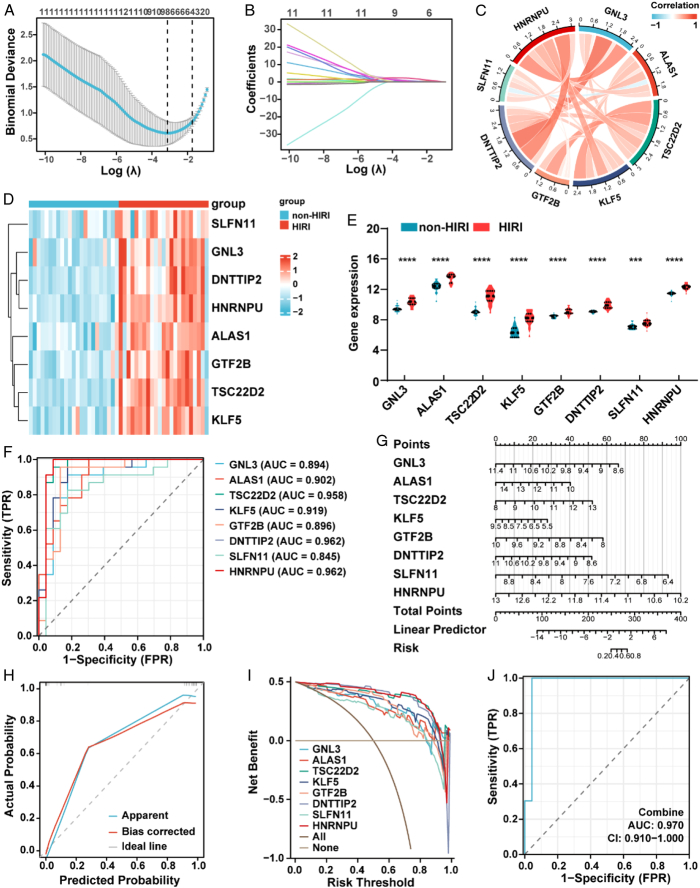
Identification of featured DE-CRGs via machine learning. (A, B) Construction of featured DE-CRGs using LASSO regression. (C) Chord diagram displaying the relationship between the overlapping cuproptosis genes. (D, E) Clustered heatmap and overall expression violin plot of 8 DE-CRGs between HIRI and non-HIRI. (F) ROC curve of 8 DE-CRGs in HIRI diagnosis. (G) Nomogram model for predicting HIRI risk based on 8 genes. (H) Construction of calibration curve. (I) Decision curve showing the clinical value of the nomogram. (J) Calibration curve of the nomogram for predicting HIRI. DE-CRGs, differentially expressed cuproptosis-related genes; HIRI, hepatic ischemia-reperfusion injury; ROC, receiver operating characteristic. ****P< 0.0001, ***P< 0.001.

As described above, there were 19 DE-CRGs in HIRI. To identify the featured markers for HIRI, we conducted LASSO analysis, which revealed that the optimal lambda value was 0.0435 after tenfold cross-validation. The regression coefficients of GNL3 (guanine nucleotide-binding protein like 3), ALAS1 (aminolevulinic acid synthase 1), TSC22D2 (transforming growth factor β stimulated clone 22 domain family, member 2), KLF5 (Krüppel-like Factor 5), GTF2B (general transcription factor IIB), DNTTIP2 (deoxynucleotidyl transferase terminal-interacting proteins 2), SLFN11 (schlafen 11) and HNRNPU (heterogeneous ribonucleoprotein-U) were 0.063, 0.222, 0.450, 0.027, 0.974, 0.517, 0.486 and 2.232, respectively (Fig. 2A, B). And the 95% CIs and *p* values of these coefficients were shown in Table S2. Subsequently, we analyzed the correlation between 8 featured DE-CRGs to explore whether cuproptosis plays a role in the development of HIRI. The results indicated that all 8 genes were highly correlated with each other, except SLFN11 and ALAS1. The correlation between GNL3 and DNTTIP2 was the strongest (correlation coefficient 0.84) (Fig. 2C). The heatmap and expression level of 8 featured DE-CRGs showed that they were all expressed at significantly higher levels in HIRI samples than in non-HIRI samples (Fig. 2D-E). To predict HIRI, the ROC curves of 8 genes were analyzed. Notably, DNTTIP2 and HNRNPU had the highest area under the curve (AUC) among the 8 genes, with a value of 0.962. Other AUC values for GNL3, ALAS1, TSC22D2, KLF5, GTF2B and SLFN11 were 0.894, 0.902, 0.958, 0.919, 0.896, and 0.845, respectively (Fig. 2F). We developed a nomogram to further evaluate the predictive effectiveness of the HIRI model (Fig. 2G). The calibration curves and clinical decision curves also indicated that the line plot was accurate and had excellent net clinical benefit (Fig. 2H, I). Similarly, the ROC-AUC of the risk score was 0.970, which is indicative of excellent model discrimination (Fig. 2J). Overall, these results indicated that all 8 featured DE-CRGs had excellent diagnostic value.

### Single-gene enrichment analysis of 8 DE-CRGs

**Figure 3:**
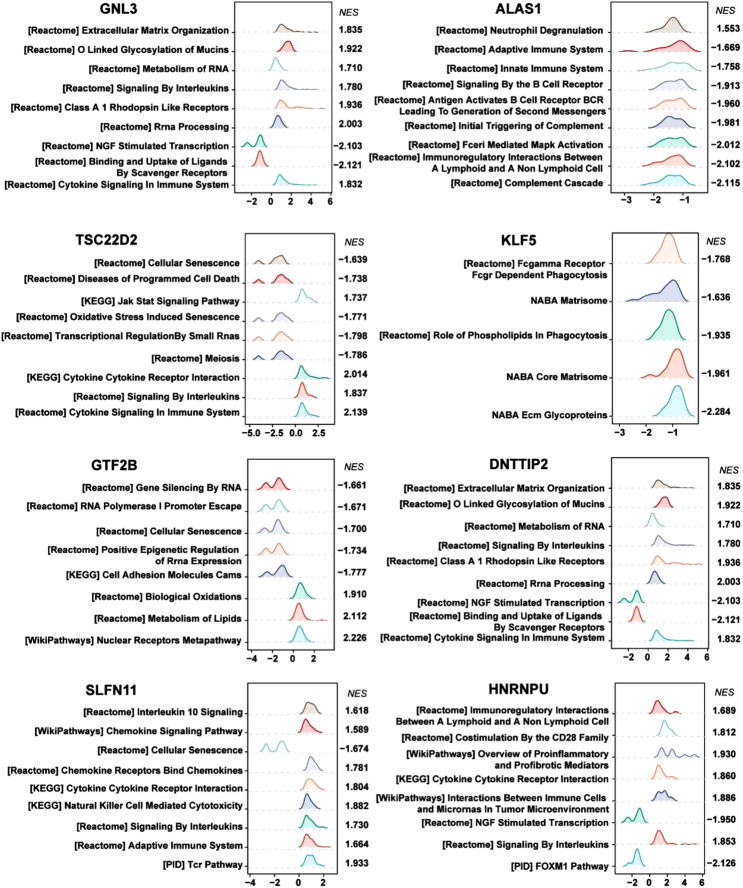
Single-gene GSEA of 8 DE-CRGs. GSEA of GNL3, ALAS1, TSC22D2, KLF5, GTF2B, DNTTIP2, SLFN11 and HNRNPU in pathways. DE-CRGs, differentially expressed cuproptosis-related genes; GSEA, gene set enrichment analysis.

Single-gene GSEA was performed to explore the potential mechanisms of 8 DE-CRGs in HIRI （Fig. 3）. Interestingly, GNL3, ALAS1, TSC22D2, DNTTIP2, SLFN11 and HNRNPU were associated with signaling by interleukins, which were all upregulated in HIRI. In addition, the signaling pathways in which GNL3, ALAS1 and TSC22D2 were upregulated were also related to cytokine signaling in the immune system. However, we found that TSC22D2, GTF2B and SLFN11 were negatively associated with cellular senescence. ALAS1, TSC22D2, GTF2B and HNRNPU were associated with the complement cascade, diseases of programmed cell death, biological oxidation and overview of proinflammatory and profibrotic mediators, respectively. Therefore, we believed that there was a strong relationship between the DE-CRGs and the immune response, oxidative stress and cell death in HIRI.

### Immune infiltration and cell death analysis of 8 DE-CRGs

GO/KEGG analysis revealed that the immune response, apoptosis and ferroptosis appeared to play roles in HIRI. Moreover, a close relationship was found between 8 DE-CRGs and immune pathways and cell death in single-gene GSEA. To further confirm the role of the 8 DE-CRGs in HIRI, we conducted immune infiltration and cell death analyses.

**Figure 4:**
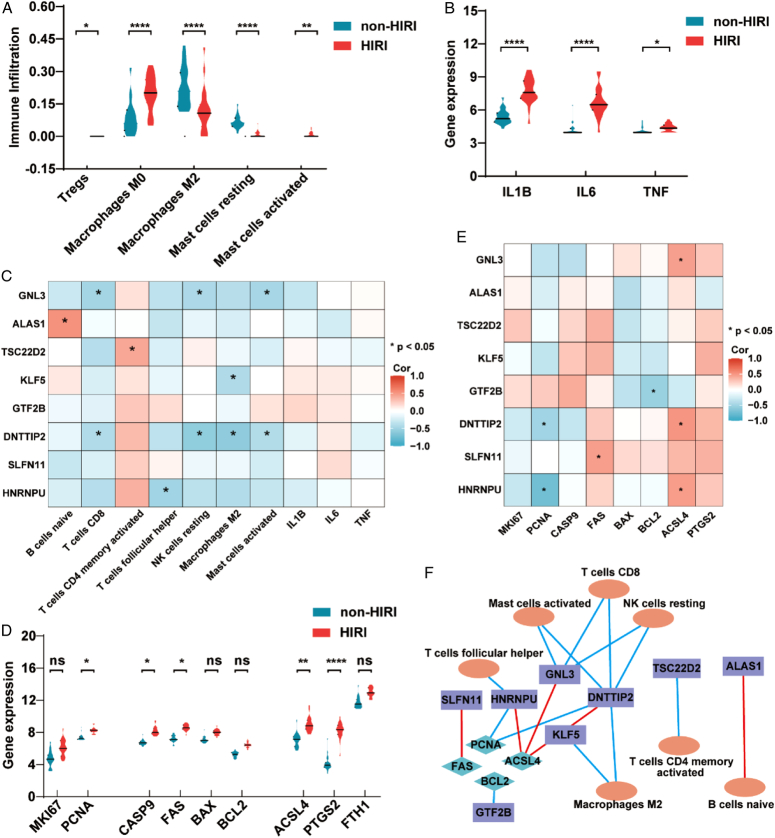
Immune infiltration and cell death analysis of 8 DE-CRGs. (A) Comparison of immune infiltration between the HIRI and non-HIRI groups. (B) Comparison of cytokines between the HIRI and non-HIRI groups. (C) Correlation analysis of 8 DE-CRGs and immune cells. (D) Comparison of cell death between the HIRI and non-HIRI groups. (E) Correlation analysis of 8 DE-CRGs and cell death. (F) Significant immune infiltration and cell death analysis among 8 DE-CRGs. Blue lines mean negative correlationship, red lines mean positive correlationship. DE-CRGs, differentially expressed cuproptosis-related genes; HIRI, hepatic ischemia-reperfusion injury. ****P< 0.0001, **P< 0.01, *P< 0.05.

The CIBERSORT algorithm calculated the relative contents of 22 immune cells in every HIRI and normal sample. The heatmap shows the normalized enrichment score of immune infiltration (Supplementary Fig 1A, Supplemental Digital Content 1, http://links.lww.com/JS9/C894). Moreover, we conducted a correlation analysis of immune cells in the HIRI group and found that the correlation between activated dendritic cells and activated NK cells was the strongest (Supplementary Fig 1B, Supplemental Digital Content 1, http://links.lww.com/JS9/C894). The results of differential analyses showed that 5 immune cells of the HIRI and non-HIRI groups were different, namely, Tregs, M0 macrophages, M2 macrophages, resting mast cells and activated mast cells (Fig. 4A). The enrichment analysis of the other cells is shown in Supplementary Fig 1C, Supplemental Digital Content 1, http://links.lww.com/JS9/C894. M2 is a suppressor of inflammation; however, its content was downregulated in the HIRI group. Therefore, we further analyzed cytokines, including IL1B, IL-6 and TNF. As shown in Fig. 4B, the levels of IL1B, IL-6 and TNF in the HIRI group were higher than those in the non-HIRI group. These results indicated that the immune response played a role in HIRI. Subsequently, we analyzed the correlation between the immune response and 8 DE-CRGs (Fig. 4C and Supplementary Fig 1D, Supplemental Digital Content 1, http://links.lww.com/JS9/C894). We found that GNL3 was negatively correlated with CD8 T cells, resting NK cells and activated mast cells. DNTTIP2 was negatively correlated with CD8 T cells, resting NK cells, M2 macrophages and activated mast cells, and KLF5 and HNRNPU were negatively correlated with M2 macrophages and follicular helper T cells, respectively. However, ALAS1 and TSC22D2 were positively correlated with naïve B cells and activated memory CD4 T cells, respectively. Similarly, the 8 DE-CRGs seemed to be negatively correlated with IL1B, IL-6 and TNF without statistical significance.

Next, we compared the differences in cell apoptosis and ferroptosis between the HIRI and non-HIRI groups. We selected MKI67 and PCNA as indicators of cell proliferation. Caspase 9, FAS, bax and bcl-2 were selected as indicators of apoptosis, and ACSL4, PTGS2 and FTH1 were selected as indicators of ferroptosis. The results indicated that cell proliferation decreased, and apoptosis and ferroptosis occurred in the HIRI group (as shown in Fig. 4D), which was shown in a previous study^28,29^. Moreover, we found that DNTTIP2 and HNRNPU were negatively correlated with PCNA, indicating that DE-CRGs inhibited cell proliferation. Moreover, GNL3, DNTTIP2 and HNRNPU were positively correlated with ACSL4, which indicated potential crosstalk between cuproptosis and ferroptosis (as shown in Fig. 4E). In all, we exported a CRGs-immune cells-cell death related network to illustrate above findings in Fig. 4F.

### HIRI unsupervised clustering identification and analysis

**Figure 5:**
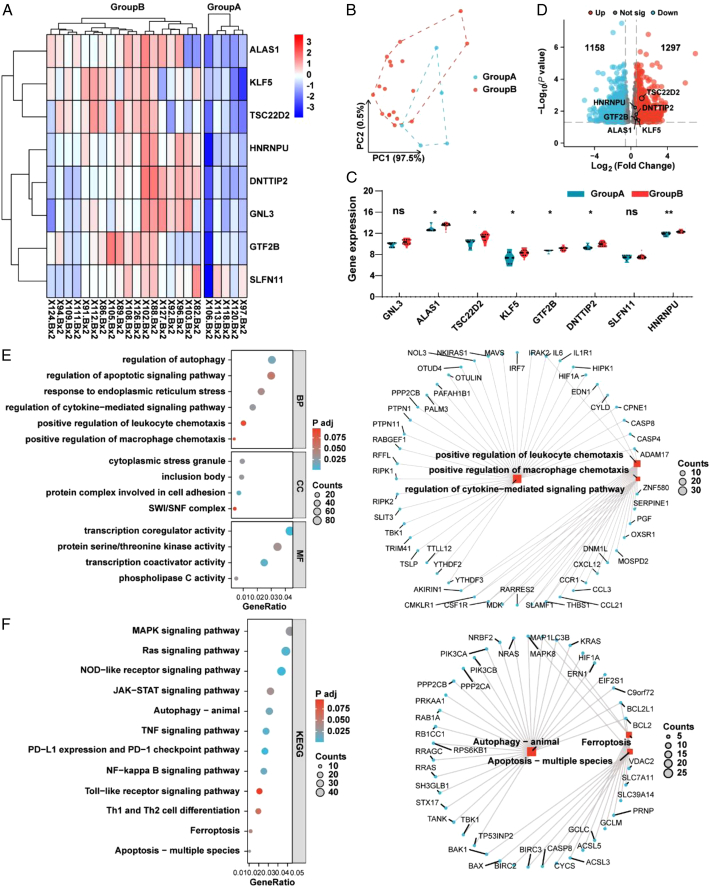
HIRI unsupervised clustering identification and analysis. (A) Expression heatmap of 8 DE-CRGs between groups A and B. (B) Principal component analysis plot of the 2 groups. (C) Expression violin plot of 8 DE-CRGs between the 2 groups. (D) Volcano plot of DEGs between the 2 groups. (E, F) GO/KEGG analysis of DEGs between the 2 groups. HIRI, hepatic ischemia-reperfusion injury; DE-CRGs, differentially expressed cuproptosis-related genes; HIRI, hepatic ischemia-reperfusion injury. **P< 0.01, *P< 0.05.

To address whether the 8 CR-DEGs can discriminate the samples within HIRI, we used a hierarchical clustering algorithm to identify 23 HIRI samples with the expression of 8 CR-DEGs. Finally, we divided the 23 HIRI samples into two groups, namely, group A (*n*=5) and group B (*n*=18) (Fig. 5A). PCA showed significant differences between the 2 groups (Fig. 5B). Differential analysis of the two groups showed that the expression of ALAS1, TSC22D2, KLF5, GTF2B, DNTTIP2 and HNRNPU in group A was lower than that in group B (Fig. 5C). In addition, we identified a total of 2455 DEGs (|log2 (FC) | >0 and *P* < 0.05), and the volcano plot is shown in Fig. 5D. We conducted a GO/KEGG enrichment analysis to explore the characteristics between the two groups. The GO results showed that the DEGs were related to the regulation of autophagy, regulation of the apoptotic signaling pathway, response to endoplasmic reticulum stress, and positive regulation of macrophage chemotaxis (Fig. 5E). As shown in Fig. 5F, DEGs were mainly enriched in the Ras signaling pathway, TNF signaling pathway, apoptosis - multiple species, and ferroptosis.

### Initial validation of 8 DE-CRGs

**Figure 6:**
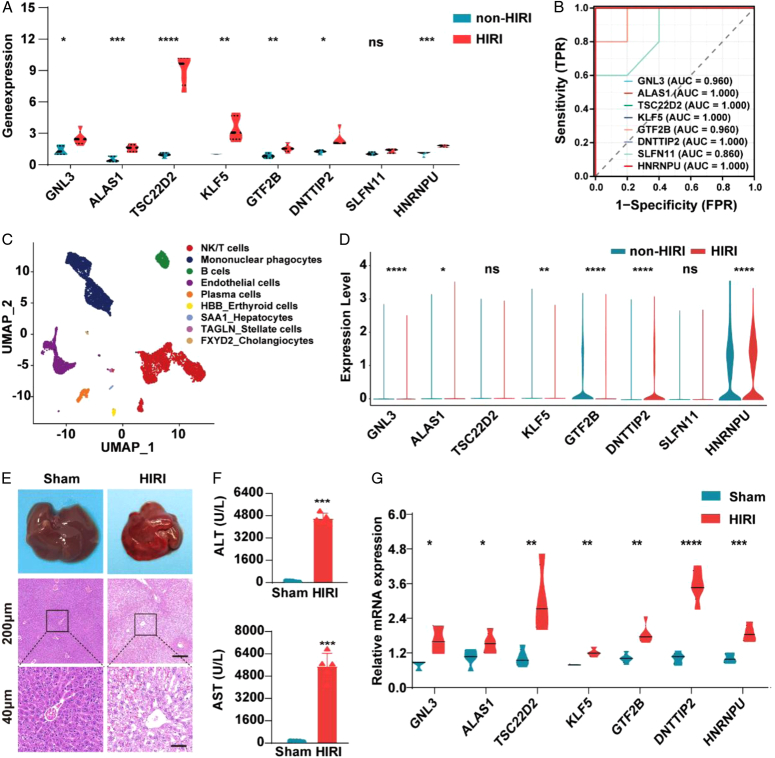
Initial validation of 8 DE-CRGs. (A) Overall expression violin plot of 8 DE-CRGs between HIRI and non-HIRI in the GSE14951 dataset. (B) ROC curve of 8 DE-CRGs in HIRI diagnosis in the GSE14951 dataset. (C) The tSNE map in the GSE171539 dataset. (D) Overall expression violin plot of 8 DE-CRGs between HIRI and non-HIRI in the GSE171539 dataset. (E). General images of mice and hematoxylin and eosin in the sham and HIRI groups. (F) Serum ALT and AST levels in the sham and HIRI groups. (G) The relative mRNA expression of 8 genes in the sham and HIRI groups. DE-CRGs, differentially expressed cuproptosis-related genes; HIRI, hepatic ischemia-reperfusion injury; ROC, receiver operating characteristic. (*N*=5 each group) ****P< 0.0001, ***P< 0.001, **P< 0.01, *P< 0.05.

Finally, we chose GSE14951 to validate the expression and diagnostic value of 8 DE-CRGs in HIRI. Similar to the results of the GSE151648 trial gene set, seven genes of the HIRI group, except SLFN11, were higher than those of the non-HIRI group (Fig. 6A). The corresponding AUC values of all 8 DE-CRGs were more than 0.86 (Fig. 6B).

Furthermore, two samples from the snRNA-seq data of GSE171539 were quality controlled and data standardized, and 8841 cells were retained for analysis. As shown in Fig. 6C, the tSNE maps demonstrated annotation of nine cell types, including NK/T cells, mononuclear phagocytes, B cells, endothelial cells, plasma cells, thyroid cells, hepatocytes, stellate cells and cholangiocytes. According to different anchor genes for all clusters, hepatocytes were annotated as SAA1 (Supplementary Fig 2A, Supplemental Digital Content 1, http://links.lww.com/JS9/C894). We first evaluated the distribution of 8 genes and found that GNL3 and GTF2B were mainly expressed in plasma cells. TSC22D2 and DNTTIP2 were mainly expressed in endothelial cells. KLF5 was mainly expressed in cholangiocytes. HNRNPU was widely expressed in all kinds of cells except hepatocytes (Supplementary Fig 2B, Supplemental Digital Content 1, http://links.lww.com/JS9/C894). Furthermore, we conducted a differential analysis between HIRI and non-HIRI and found that GNL3, KLF5, DNTTIP2 and HNRNPU were more highly expressed in HIRI samples than in non-HIRI samples. However, ALAS1 and GTF2B had lower expression in HIRI than in non-HIRI. There were no significant differences in TSC22D2 and SLFN11 (Fig. 6D). Of course, the dataset included only 64 hepatocytes, which was the largest cell population in the liver. Therefore, we need to enlarge the sample size to support the above results. To further validate our findings, we established an HIRI mouse model (Fig. 6E-F). Consistent with our above results, the mRNA levels of the seven genes (SLFN11 excluded) were significantly upregulated in HIRI mice compared to sham mice (Fig. 6G), indicating that GNL3, ALAS1, TSC22D2, KLF5, GTF2B, DNTTIP2, SLFN11 and HNRNPU may be involved in HIRI.

**Figure 7:**
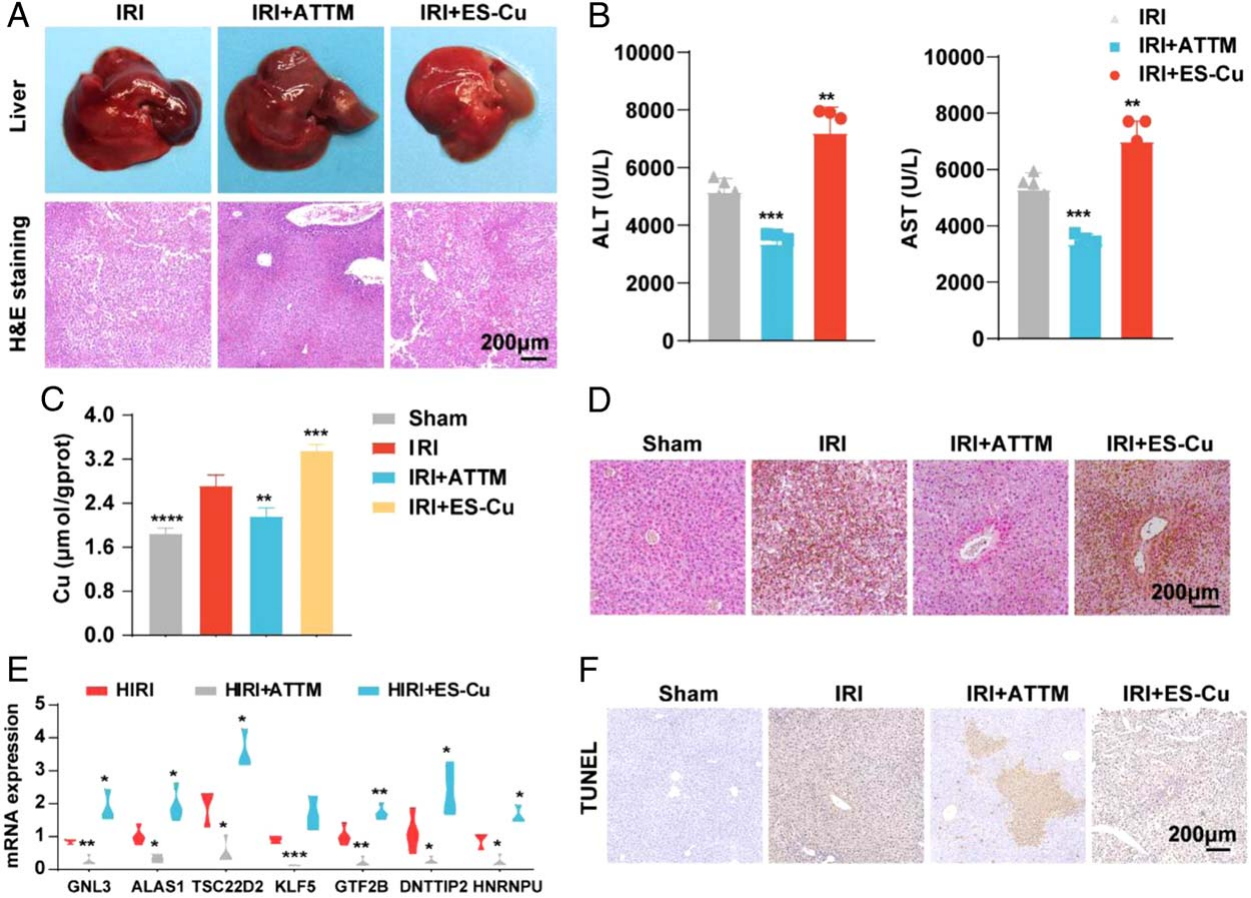
The occurrence of copper death and the crosstalk with apoptosis in HIRI. (A) General images of mice and hematoxylin and eosin after the administration of cuproptosis inducers and inhibitors. (B) Serum alanine aminotransferase (ALT) and aspartate transaminase (AST) levels after the administration of cuproptosis inducers and inhibitors. (C). The copper content in liver. (D). The copper rubethine stain in liver. (E). The relative mRNA expression of 8 genes. (F) The tunel stain in liver. HIRI, hepatic ischemia-reperfusion injury. (*N*=5 each group) ***P< 0.001, **P< 0.01, *P< 0.05.

To verify the occurrence of copper death and the crosstalk with apoptosis in HIRI, we added cuproptosis inducers and inhibitors to treat HIRI mice again. We found that cuproptosis inhibitors improved liver damage, but cuproptosis inducers showed the opposite effect (Fig. 7A-B). Furtherly, we found that the copper content of HIRI mice increased, and the administration of inhibitors and inducers affected the copper content (Fig. 7C-D). And cuproptosis inhibitors decreased the expression of 7 genes, but the inducers increased the expression (Fig. 7E). Finally, we found cuproptosis inhibitors could reduce the occurrence of apoptosis, which confirmed the crosstalk between copper death and apoptosis (Fig. 7F). Taken together, these findings support that 8 DE-CRGs are key players in HIRI.

### Construction of a transcription factor-DE-CRGs network and a miRNA-DE-CRGs network

**Figure 8:**
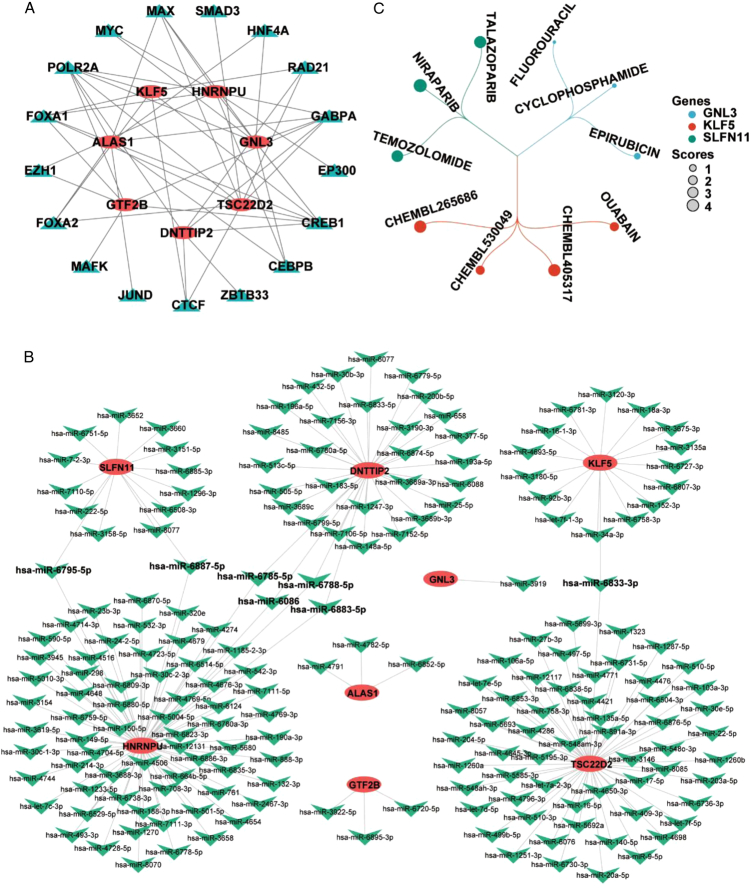
The network of TF-DE-CRGs, miRNA-DE-CRGs and drug interaction. (A) The TFs prediction on 8 DE-CRGs. (B) The miRNA prediction on 8 DE-CRGs. (C) Prediction of drug-gene interactions on 8 DE-CRGs. DE-CRGs, differentially expressed cuproptosis-related genes; TF, transcription factors.

To further explore the complex molecular interaction mechanism between miRNAs/TFs and 8 DE-CRGs, we created a miRNA-mRNA and TF-mRNA network relationship diagram, as shown in Fig. 8A-B. The TF-mRNA complex network showed 17 TFs might regulate the mRNA of DE-CRGs. SLFN11 was excluded because of its prediction was created in 1 database. POLR2A and CREB1 were TFs that played the most regulatory role. We also predicted miRNAs capable of interacting with 8 DE-CRGs. The miRNA-DE-CRGs interaction network with a total of 192 miRNAs, and HNRNPU shared 2 and 4 miRNAs with SLFN11 and DNTTIP2, respectively. And GNL3 has only a miRNA to interact. The 8 DE-CRGs had multiple binding sites for TFs and miRNAs, which could guide our further studies on their mechanism.

### Prediction of drug-gene interactions

A total of 10 drugs that might have regulatory relationships with the DE-CRGs were screened, among which the number of drugs interacting with KLF5 was the largest (Fig. 8C). However, we finally got drug predictions for 3 DE-CRGs, including GNL3, KLF5 and SLFN11. We’re going to get more drug predictions as research develops.

## Discussion

Liver transplantation is the only effective treatment for liver cancers and end-stage liver diseases. However, HIRI is a critical complication and the primary risk factor for early allograft failure after LT, which also results in the shortage of organs for transplantation^30^. Repressing HIRI would allow for the usage of marginal allografts and improve clinical outcomes. Currently, strategies for blocking HIRI involve clearing ROS, blocking immune activation and regulating the cytokine response^31^. Thus, an increasing number of studies have focused on the cellular and molecular mechanisms of HIRI. Cuproptosis is a copper-dependent form of cell death that is mainly caused by the aggregation of lipid acylation-related proteins and the loss of iron-sulfur cluster proteins. Cuproptosis occurs in the brain I/R process and cardiovascular disease^32,33^. However, the association between cuproptosis and HIRI is unclear. Bioinformatics is widely used to analyze genome sequencing data and gain meaningful biological information. The current study conducted data mining and machine learning to determine the relationship between cuproptosis-related genes and HIRI phenotypes and to analyze the role of these genes in immune infiltration. We further predicted subgroups of HIRI based on DE-CRGs. Finally, the DE-CRGs were verified in the HIRI model by qRT–PCR.

First, we found specific genes that were significantly different between pre-ischemia and post-ischemia based on the GSE151648 dataset. A total of 2390 DEGs were identified, and pathway enrichment analysis revealed that these DEGs were associated with the inflammatory response, response to reactive oxygen species, regulation of metal ion transport, apoptosis, ferroptosis and HIF-1 signaling pathway. Studies have found that inflammatory cell infiltration and inflammatory mediators, hepatocyte necrosis and apoptosis caused by free radicals were the key pathophysiological changes in HIRI^3^. This is consistent with our primary findings. Moreover, 19 DE-CRGs were defined in HIRI, and these DE-CRGs intersected with DEGs.

We obtained eight featured DE-CRGs using LASSO regression analysis, including GNL3, ALAS1, TSC22D2, KLF5, GTF2B, DNTTIP2, SLFN11 and HNRNPU. The expression levels of all these genes were higher in HIRI samples than non-HIRI samples and had good diagnostic values in HIRI. GNL3 is a member of the MMR1/HSR1CTP-binding protein family and is involved in the proliferation cycle of stem cells and tumor cells^34^. ALAS1 is the rate-limiting enzyme of heme synthesis in the liver and is upregulated in acute hepatic porphyrias^35^. Lian *et al*.^36^ also found that ALAS1 was essential for neutrophil maturation in zebrafish. TSC22D2 is a novel cancer-associated gene in a rare multicancer family, and high TSC22D2 expression is an independent predictor for the poor prognosis of pancreatic adenocarcinoma^37^). KLF5 participates in cell proliferation, differentiation, and migration and has been found to be involved in inflammatory bowel disease, airway inflammation and tumor immunity^38–40^. GTF2B is a transcription factor that is involved in hepatocellular carcinoma and growth hormone-secreting pituitary adenoma^41^. DNTTIP2 is a regulator of terminal deoxynucleotidyl transferase and plays a role in transcriptional regulation. Liu found that DNTTIP2 affected macrophage M2 activation and promoted angiogenesis in gliomas^42^. SLFN11 is a putative DNA/RNA helicase and is linked to the antiviral response in human cells and interferon production. Recent studies have found that SLFN11 plays a role in various cancers^43^. HNRNPU is a nuclear scaffold protein that regulates lung inflammation, antiviral responses and aberrant differentiation of Tfh cells^44–46^.

Next, we conducted GSEA for all 8 DE-CRGs in HIRI and found that most of them were involved in the immune response. TSC22D2, GTF2B and SLFN11 were negatively associated with cellular senescence. Therefore, we further conducted immune infiltration and cell death analysis of 8 DE-CRGs. We found that Tregs, M0 macrophages and activated mast cells were upregulated in HIRI; however, M2 macrophages and resting mast cells were downregulated in HIRI. Moreover, the expression of IL1B, IL-6 and TNF was higher in HIRI. As previously described, the dysregulation of hepatic microcirculation and mitochondrial function causes very early-phase injury and initiates an immune cascade in HIRI. Subsequent immunity prolongs liver injury through recruitment of macrophages, neutrophils, and dendritic cells and by activation of ILCs, NKT cells, and cytotoxic T lymphocytes^6^. M2 macrophages are anti-inflammatory cells that inhibit T-cell proliferation and activation and secrete IL-10, IL-4, and transforming growth factor-β. Here, our findings indicated that there were serious immune responses in HIRI, which was consistent with previous studies. Moreover, we found that GNL3 was negatively associated with CD8 T cells and resting NK cells. DNTTIP1 was negatively associated with CD8 T cells, resting NK cells and M2 macrophages, and KLF5 was negatively associated with M2 macrophages. However, ALAS1 and TSC22D2 were positively associated with naïve B cells and activated memory CD4 T cells, respectively. In all, we found that most of the 8 DE-CRGs had a negative relationship with immune infiltration, which was driven by complicated interactions among different immune cell types.

Apoptosis is the crucial cell death pathway in HIRI. However, a mixture of death signaling pathways, including necroptosis, pyroptosis and ferroptosis, are involved in HIRI^6,47^. Because of the diversity of hepatic cell death programs in HIRI, there are always complementary or overlapping forms of regulated cell death^47^. We found that cell proliferation was downregulated, and apoptosis and ferroptosis were upregulated based on featured markers in HIRI. Moreover, we found hepatocytes apoptosis was positively correlated with the addition of cuproptosis inducers and inhibitors in HIRI mice. Also, the genes associated with ferroptosis were significantly changed in HIRI. Moreover, DNTTIP2 and HNRNPU were negatively associated with PCNA, and GNL3, DNTTIP2 and HNRNPU were positively associated with ACSL4. ACSL4 (acyl-CoA synthetase long-chain family member 4) is involved in various biological processes by regulating lipid metabolism and is a crucial factor in ferroptosis^48^. Therefore, we concluded that there may be a crosstalk between cuproptosis and other cell death in HIRI.

Based on the eight DE-CRGs, we utilized hierarchical clustering analysis to identify two distinct cuproptosis-related clusters. In group B, the expression of six DE-CRGs was significantly higher than that in group A (except GNL3 and SLFN11). In the subcluster function analysis, we found that the DEGs were primarily enriched in the regulation of autophagy, response to endoplasmic reticulum stress, regulation of apoptotic signaling pathway, TNF signaling pathway and ferroptosis. Overall, these results reflected a stronger immune response and cell damage in HIRI with eight higher DE-CRGs.

To further confirm the reliability of the diagnostic model, we performed dataset and mouse model validation. We found that seven DE-CRGs (except SLFN11) were higher in the HIRI group based on GSE14951, and GNL3, KLF5, DNTTIP2 and HNRNPU had higher expression in the HIRI group than in the non-HIRI group based on GSE171539. However, GSE171539 offered data from 2 samples and 64 hepatocytes. Therefore, the single-cell expression of the other four DE-CRGs need to be further verified. Finally, we verified that the expression of seven DE-CRGs (SLFN11 excluded) was significantly upregulated in the HIRI mouse model. They showed decreased or increased after the administration of cuproptosis inhibitors or inducers. The copper content decreased or increased after the addition of cuproptosis inhibitors or inducers. In conclusion, the systematic study of diagnostic markers of HIRI will help us better understand the pathogenesis of HIRI and provide a theoretical basis for its personalized diagnosis and treatment.

Finally, we explored the potential regulatory mechanisms of DE-CRGs and predicted some therapeutic drugs for 8 DE-CRGs. However, these findings are predictive and need to be further verified by wet-trials.

Nonetheless, this study has several limitations. First, our results need to be further demonstrated using clinical samples. Additionally, we may construct a more precise nomogram for prognosis by integrating the model with complications, sex, age, etc. Second, there may be unavoidable selection bias because the data were mined from based on a few public datasets base, and the sample size of each was still relatively small. We need more HIRI subjects to validate our findings. In addition, the cellular and molecular functions and HIRI-related mechanisms of these eight DE-CRGs need to be further explored through wet-bench experiments in vitro and in vivo.

## Conclusion

In conclusion, we screened eight signature genes (GNL3, ALAS1, TSC22D2, KLF5, GTF2B, DNTTIP2, SLFN11 and HNRNPU) associated with HIRI and cuproptosis through a series of bioinformatics analyses. Additionally, our work indicated an association between DE-CRGs and immune infiltration, as well as the heterogeneity among subgroups of HIRI patients. The optimal machine learning model based on the eight DE-CRGs can accurately predict the risk of HIRI occurrence and assess HIRI subtypes. Moreover, we constructed a miRNA/TFs network and predicted the gene-targeted drugs. Therefore, our study provides new insights into the clinical heterogeneity and prognosis of HIRI and provides a theoretical basis for the underlying pathophysiological process of HIRI and potential HIRI treatments in the future.

## Ethical approval

Mouse experiments were performed in accordance with a protocol approved by the Second Affiliated Hospital of Zhejiang University School of Medicine (Ethics Committee number: 105, year 2023).

## Consent

Not applicable.

## Source of funding

This research was supported by the Key Research and Development Program of Zhejiang Province (No. 2021C03121), National Natural Science Foundation of China (No. 82302900 and 82072650) and Zhejiang University Basic Research Fund (No. 226-2022-00037).

## Author contribution

All authors contributed to the study’s conception and design. Material preparation and data collection were performed by K.W., H.C., Y.Y., J.J., J.Y., J.G., Y.Z. Data analysis was performed by X.C., J.D., X.Z., H.C. The first draft of the manuscript was written by X.C., J.D. and reviewed by X.C., Y.D., Q.S., W.W. The manuscript was supervised and finalized by X.C. and W.W. All authors read and approved the final manuscript.

## Conflicts of interest disclosure

The authors declare that they have no competing interests.

## Research registration unique identifying number (UIN)

Not applicable.

## Guarantor

Xiaopeng Cai and Weilin Wang.

## Availability of data and materials

The datasets generated during and/or analyzed during the current study are available from the corresponding author on reasonable request.

## Provenance and peer review

Not applicable.

## Supplementary Material
