# Supplementary material for: Comprehensive analysis of cuproptosis-related genes involved in immune infiltration and their use in the diagnosis of hepatic ischemia-reperfusion injury: an experimental study
Source: Int J Surg. 2024 Jun 27;111(1):242–56. doi: 10.1097/JS9.0000000000001893 (PMC11745764; doi:10.1097/JS9.0000000000001893)
Supplement: Supplementary file 2 [file js9-111-0242-s002.docx]

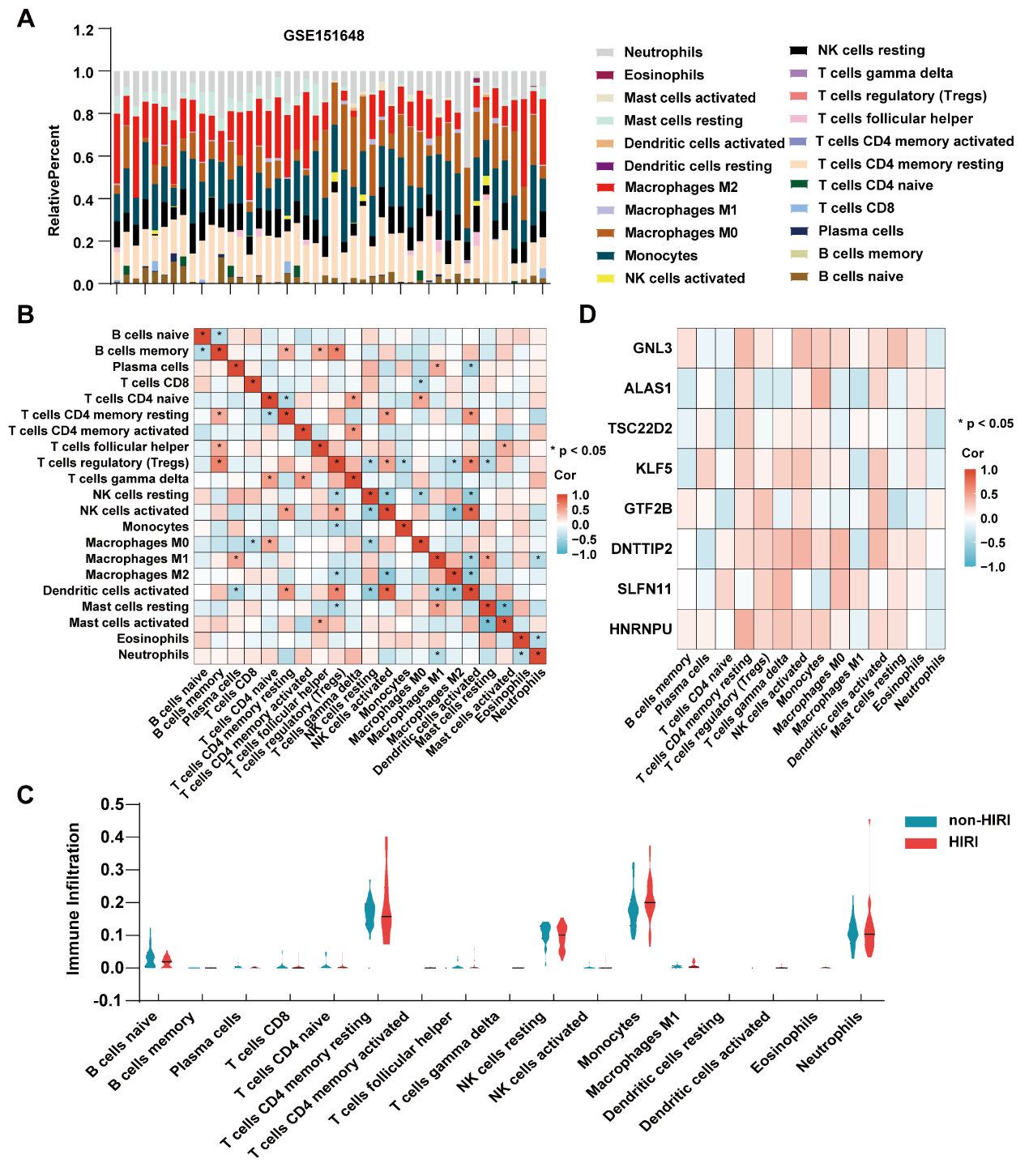


Supplementary figure 1. Immune infiltration analysis of 8 DE-CRGs. A. Relative abundance of infiltrating immune cells in the HIRI and non-HIRI groups. B. The relationship among infiltrating immune cells. C. Comparison of immune infiltration between the HIRI and non-HIRI groups. D. Correlation analysis between 8 DE-CRGs and immune cells. DE-CRGs, differentially expressed cuproptosis-related genes. *p< 0.05.


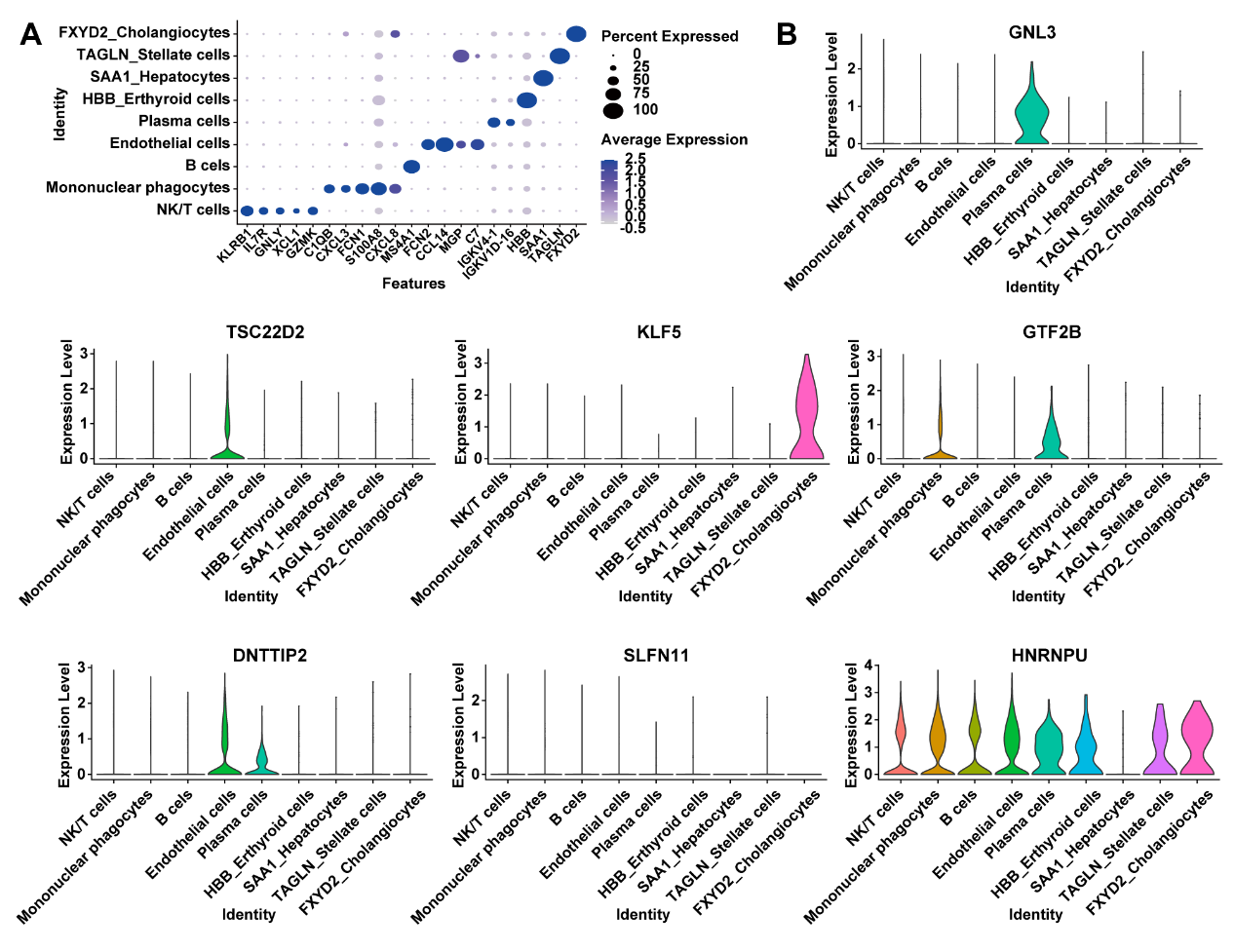


Supplementary figure 2. Initial validation of 8 DE-CRGs. A. The dot plot displaying the expression patterns and the percentages of markers in each enriched cluster in the GSE171539 dataset. B. The distribution of 8 genes in the GSE171539 dataset.
